# Neuropathology and Inflammatory Cell Characterization in 10 Autoptic COVID-19 Brains

**DOI:** 10.3390/cells10092262

**Published:** 2021-08-31

**Authors:** Daniele Colombo, Laura Falasca, Luisa Marchioni, Antonella Tammaro, Ganiyat Adenike Ralitsa Adebanjo, Giuseppe Ippolito, Alimuddin Zumla, Mauro Piacentini, Roberta Nardacci, Franca Del Nonno

**Affiliations:** 1 Pathology Unit, National Institute for Infectious Diseases “Lazzaro Spallanzani”—IRCCS, 00149 Rome, Italy; daniele.colombo@inmi.it (D.C.); franca.delnonno@inmi.it (F.D.N.); 2 Laboratory of Electron Microscopy, National Institute for Infectious Diseases “Lazzaro Spallanzani”—IRCCS, 00149 Rome, Italy; mauro.piacentini@uniroma2.it (M.P.); roberta.nardacci@inmi.it (R.N.); 3 Clinical Department, National Institute for Infectious Diseases “Lazzaro Spallanzani”—IRCCS, 00149 Rome, Italy; marchioni.luisa@inmi.it; 4 NESMOS Dermatology Department, University of Rome “La Sapienza”, 00189 Rome, Italy; antonella.tammaro@uniroma1.it (A.T.); g.adenikeadebanjo@gmail.com (G.A.R.A.); 5 Scientific Direction, National Institute for Infectious Diseases “Lazzaro Spallanzani”—IRCCS, 00149 Rome, Italy; giuseppe.ippolito@inmi.it; 6 Department of Infection, Division of Infection and Immunity, University College London and NIHR Biomedical Research Centre, UCL Hospitals NHS Foundation Trust, London W1T 7DN, UK; a.i.zumla@gmail.com; 7 Department of Biology, University of Rome “Tor Vergata”, 00133 Rome, Italy

**Keywords:** SARS-CoV-2, COVID-19, neuropathology, brain autopsy, microvascular injury

## Abstract

COVID-19 presents with a wide range of clinical neurological manifestations. It has been recognized that SARS-CoV-2 infection affects both the central and peripheral nervous system, leading to smell and taste disturbances; acute ischemic and hemorrhagic cerebrovascular disease; encephalopathies and seizures; and causes most surviving patients to have long lasting neurological symptoms. Despite this, typical neuropathological features associated with the infection have still not been identified. Studies of post-mortem examinations of the cerebral cortex are obtained with difficulty due to laboratory safety concerns. In addition, they represent cases with different neurological symptoms, age or comorbidities, thus a larger number of brain autoptic data from multiple institutions would be crucial. Histopathological findings described here are aimed to increase the current knowledge on neuropathology of COVID-19 patients. We report post-mortem neuropathological findings of ten COVID-19 patients. A wide range of neuropathological lesions were seen. The cerebral cortex of all patients showed vascular changes, hyperemia of the meninges and perivascular inflammation in the cerebral parenchyma with hypoxic neuronal injury. Perivascular lymphocytic inflammation of predominantly CD8-positive T cells mixed with CD68-positive macrophages, targeting the disrupted vascular wall in the cerebral cortex, cerebellum and pons were seen. Our findings support recent reports highlighting a role of microvascular injury in COVID-19 neurological manifestations.

## 1. Introduction

Infection with the severe acute respiratory syndrome coronavirus 2 (SARS-CoV-2) mainly affects pulmonary and cardiovascular systems, although parenchymal damage has been reported in most organs [1]. The majority of COVID-19 patients present with neurological clinical manifestations that can occur during acute infection, but can also persist after viral elimination or even emerge after viral clearance, by inducing chronic neurological sequelae. Neurological symptoms can range from headache, loss of smell and/or taste to delirium, confusion, agitation, psychosis, apathy, memory impairment, vomiting, disabling strokes, gait disorders to breathing difficulties and coma, suggesting the involvement of several brain regions [2,3,4].

The underlying mechanisms of neurological manifestations are still unclear. A direct viral involvement has not been proven, since both virus RNA and viral antigens were rarely detected in brain tissue. Whether the neuropathological manifestations are caused by infection, inflammatory or metabolic mechanisms is still a matter of research. Studies on brains post-mortem examinations are limited due to laboratory safety concerns, thus, systematic autoptic investigations are needed for accurate detection and adequate management of these patients [5,6]. A spectrum of neuropathological changes have been observed, including severe hypoxic and hemorrhagic damage, thrombotic complications, encephalitis and meningitis [7,8]. Recently, microvascular injury and neuroinflammation, with activation of innate and adaptive immune cells, have been described through histopathological studies [9,10]. Nevertheless, the role of immune activation in COVID-19 brains is debated, based on differing observations from a few small case series.

In this manuscript we describe the post-mortem neuropathological findings of ten Italian COVID-19 patients. A complete histopathological analysis was performed on the cerebral cortex, cerebellum and pons tissues, including the characterization of the observed perivascular inflammatory cells.

## 2. Materials and Methods

### 2.1. Study Cohort and Clinical Information

Cases included in this study were 10 COVID-19 patients, extracted from case records of patients who died and underwent complete post-mortem examination at the National Institute for Infectious Diseases Lazzaro Spallanzani-IRCCS Hospital in Rome, Italy.

### 2.2. Autopsy Procedures

A complete post-mortem examination was performed on 10 COVID-19 patients, in which positivity was confirmed by SARS-CoV-2 qualitative reverse transcriptase-polymerase chain reaction (RT-PCR) performed on nasopharyngeal and oropharyngeal swabs.

Autopsies were performed according to the specific guidance for post-mortem and collection of specimens and biosafety practices [11], in order to reduce the risk of transmission of infectious pathogens during and after post-mortem examination. Autopsies were performed by a team of highly trained personnel in a negative pressure biosafety level 3 (BSL-3) room, with airflow control and airborne infection control procedures including use of appropriated PPE (i.e., NIOSH-certified disposable N-95 and HEPA respirators, jumpsuit, impermeable gown with full sleeve coverage, double surgical gloves and boots). The skull opening procedure was performed with a manual saw, therefore avoiding aerosol generated from an oscillating saw, a safe alternative for skull and cerebral cortex removal procedures on suspected or confirmed COVID-19 patients.

The cerebral cortex, cerebellum and pons were separated from the cerebral hemispheres. The cerebral cortex was sliced in a coronal plane at 1 cm intervals from the frontal to the occipital lobe. The cerebellum was dissected in two halves in a horizontal plane. A portion of the frontal and temporal poles, cerebellum, pons and medulla were promptly cryopreserved at −80 °C.

### 2.3. Histological and Immunohistochemical Analysis

Cerebral samples were immediately fixed in neutral-buffered-formalin and stored for 72 h before inclusion into paraffin blocks. Sections of tissues (4 μm) were stained with hematoxylin and eosin (H&E) for histopathological analysis. Deparaffinized and rehydrated sections were used for immunohistochemistry. Sections were immersed in 10 mM sodium citrate, pH 6.0 and microwaved for antigen retrieval and immunostained on BenchMark ULTRA system fully automated instrument (Roche), with an antibody directed against CD3 (Ventana, 2GV6), CD4 (Ventana, SP35), CD8 (Ventana SP57), CD68 (Ventana, KP-1), neurofilament (Ventana, 2F11) and GFAP (Ventana EP672Y). All cases were independently analyzed by two pathologists.

Quantification of the number of perivascular CD4, CD8 and CD68 positive cells was evaluated for each patient in the cerebral cortex and pons areas. A minimum of 20 cross sectioned capillaries were analyzed per each case, and the number of perivascular inflammatory cells was counted. Cell counting was completed by 3 independent individuals; data are presented as mean ± standard deviation (SD).

## 3. Results

### 3.1. Patient Demographics and Clinical Characteristics

Clinical features, neurological history and post-mortem diagnosis of patients are summarized in Table 1.

All patients were white Caucasians, with COVID-19 confirmed by an ante-mortem polymerase chain reaction (RT-PCR) test for SARS-CoV-2 on nasopharyngeal swabs (NP). During hospitalization, three patients were treated with invasive mechanical ventilation through tracheal intubation, and one of them received extracorporeal membrane oxygenation (ECMO) for refractory hypoxemia. The other patients (except one who did not receive any therapy because of sudden death) were treated with non-invasive mechanical ventilation. The mean hospitalization time before death was 28.8 days (0–72). Laboratory parameters and clinical characteristics of patients before death are reported in Supplementary Tables S1 and S2.

### 3.2. Autopsy Macroscopic Examination

Brain gross findings of patients are summarized in Table 2.

**Figure 1:**
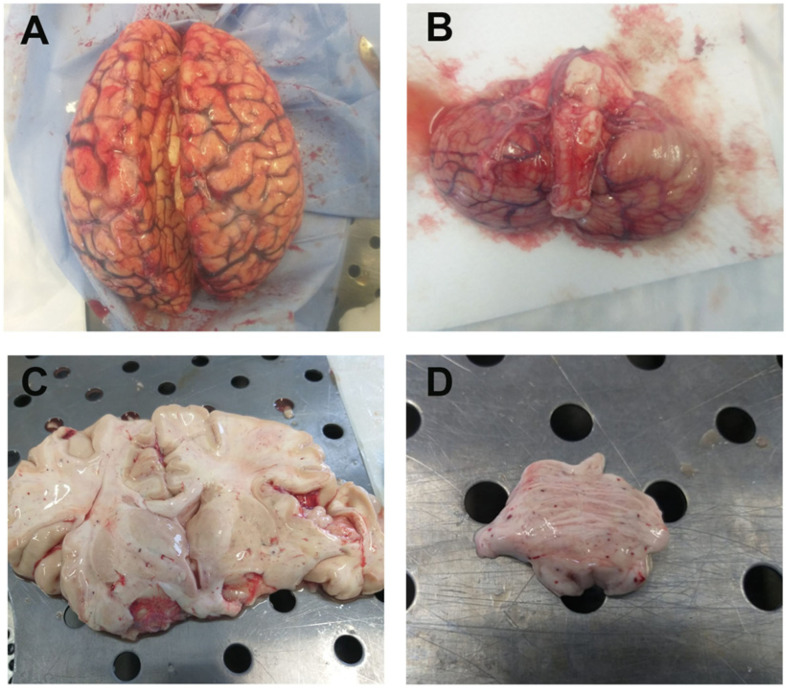
Brain gross anatomy in COVID-19 patients. (**A**) The cerebral hemispheres appear symmetrical, diminished in consistency with hyperemia of leptomeninges. Cerebral cortex parenchymal edema, macroscopically visible because of gyral flattening and compression of the sulci with broadening of perivascular spaces. (**B**) Cerebral cortex trunk in axis, midcerebral cortex and cerebellum free from macroscopically detectable alterations. (**C**) Cerebral cortex section along the coronary plane shows modest atrophy of the cerebral cortex. Shiny white substance in the cerebral cortical and basal nuclei, due to edema and hemorrhagic petechial areas. (**D**) Petechial areas in the pontine region that shows mottled appearance due to the grey discoloration. Images from patients who did not have previous neurological deficits.

Macroscopically, the cerebral cortex of 9 out of 10 patients showed hyperemia of the meninges (Figure 1A,B), and two patients showed sub-dural hematoma. In two patients, the right cerebellar hemisphere appeared hemorrhagic. In one of them, we also found a hemorrhagic lesion in the pontine area, the hematoma extended upward into the midcerebral cortex tegmentum and continued into the right thalamic nucleus. Hemorrhagic petechial areas were seen in the right frontal sub-cortical white matter of one patient, in the basal nuclei (Figure 1C) of three patients and in pons region of one patient (Figure 1D). In one patient (treated with ECMO support), there was hemorrhage in the right occipito-parietal lobes with mesencephalic hemorrhage, complicated by a collection of blood within the ventricular system.

### 3.3. Microscopic Findings

The histological examination of COVID-19 patients revealed a wide range of neuropathological lesions, highlighted both in patients with neurological degenerative diseases and in patients previously neurologically normal (Table 3). Representative images showed in the manuscript were taken from patients who did not have previous neurological deficits.

**Figure 2:**
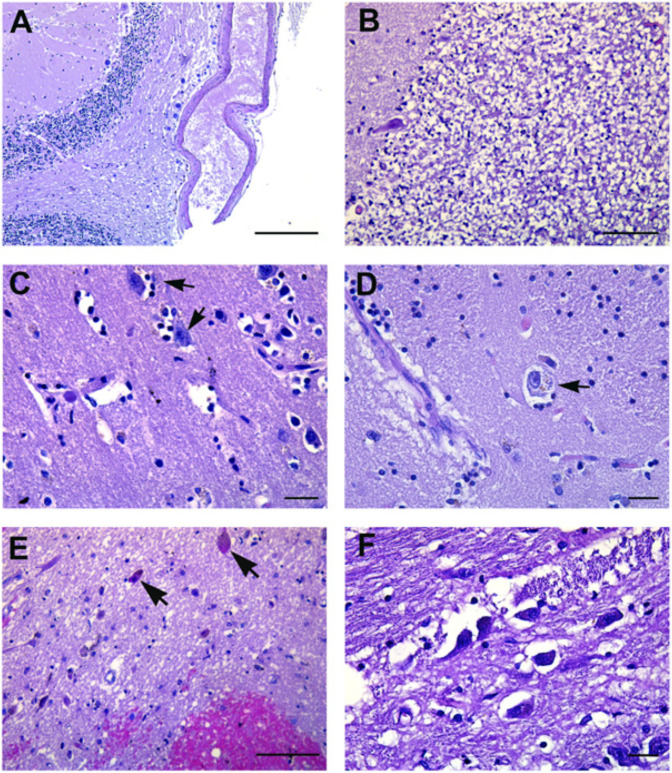
Pathological findings in neurons. (**A**) Cerebellar Purkinje cell layer, showing loss of neurons. (**B**) Disappearance of neurons in the cerebellar internal granular cell layer. (**C**,**D**) Activated resident glia with varying degrees of neuronal damage (arrows). (**E**) Pons region showing neuronal degeneration represented by small eosinophilic neurons with pyknotic nuclei (arrows). Visible is also hemorrhage around small vessels and reactive gliosis. (**F**) Pons histological section showing gray matter derangement. Images from patients who did not have previous neurological deficits. Scale bars: (**A**) = 100 µm; (**B**,**E**) = 50 µm; (**C**,**D**,**F**) = 7 µm.

Findings similar to the so-called Hypoxic-Ischemic-Encephalopathy syndrome were found. Loss of neurons was found in the cerebellar Purkinje cell layer (Table 3; Figure 2A,B).

In the pons and medulla oblongata, damaged neurons were associated with perivascular hemorrhage and gray matter derangement with vacuolization of neuropil in all cases (Figure 2C–F).

**Figure 3:**
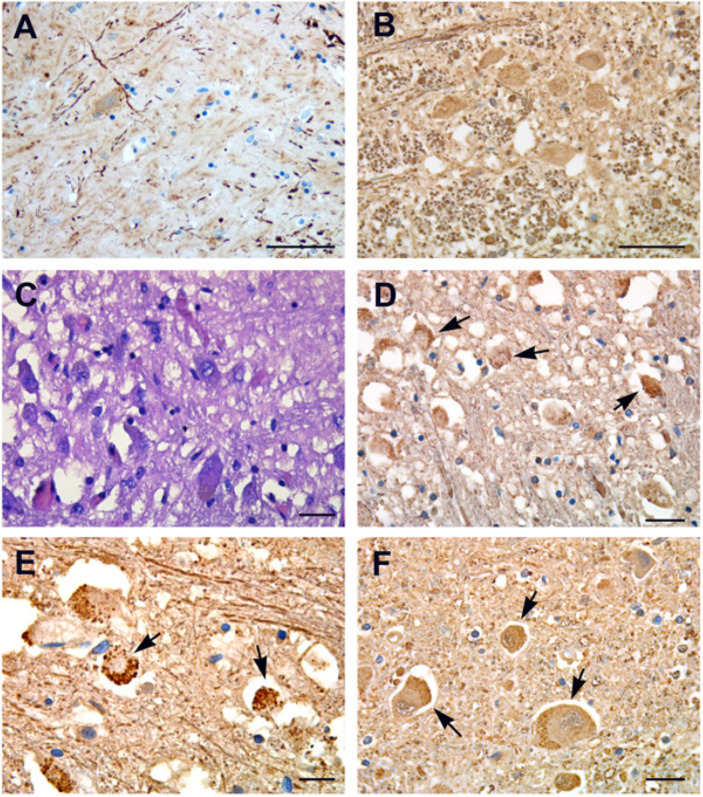
Neurofilament immunohistochemistry. (**A**,**B**) Neurofilament immunohistochemistry labels axonal spheroid, which are thick and curved axons showing axonal swelling and derangement of neuropil in basal nuclei (**A**) and in medulla oblongata (**B**). (**C**) H&E stained section showing axonal swelling and neuronal degeneration in pontine areas. (**D**–**F**) Anti-neurofilament immunolabeling of pons sections display the accumulation of small round granules in the cytoplasm of vacuolized neurons (arrows). Images from patients who did not have previous neurological deficits. Scale bars: (**A**,**B**) = 50 µm; (**C**,**E**,**F**) = 7 µm; (**D**) = 14 µm.

Neurofilaments immunolabeling showed thickened and curved axons with axonal swelling and axonal “spheroids” (Figure 3A,B). Small round granules were detected in the cytoplasm of neurons (Figure 3C–F), similar to the pre-tangle granular pattern seen in Pick’s disease or other dementias.

**Figure 4:**
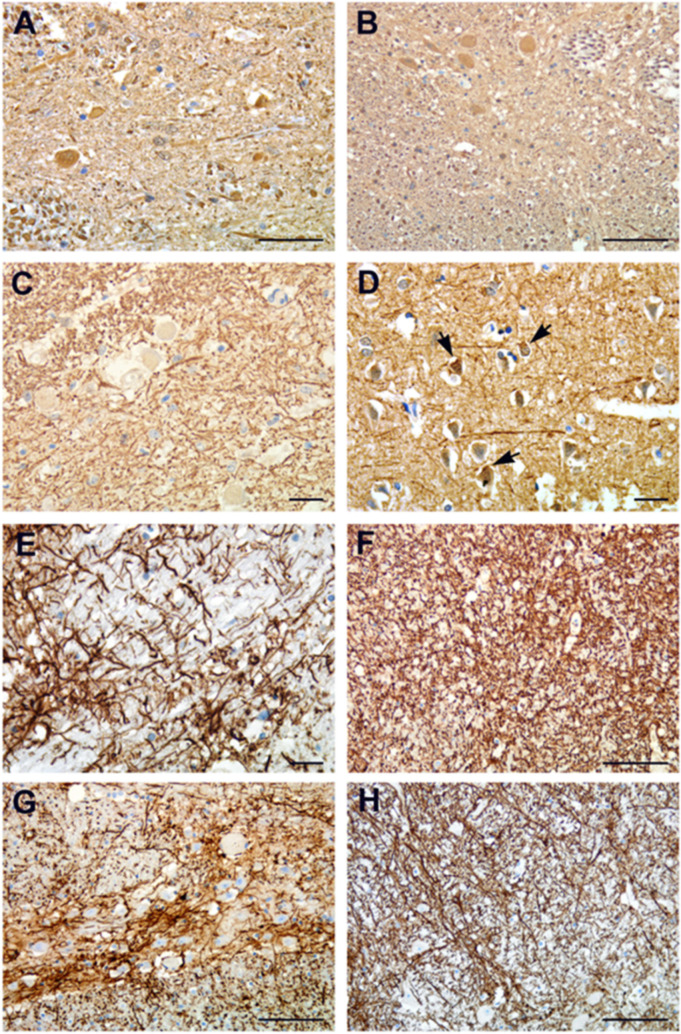
Neurofilament and GFAP immunohistochemistry. (**A**–**C**) Neurofilament protein immunohistochemistry shows neuron’s feltwork (neuropil) creating a chaotic meshwork with smaller dot-like structures. (**D**) Vacuolized and piknotic neurons (arrows). (**E**) Pathologic reactions of astrocytes highlighted by GFAP immunoreactivity showing fibrillary gliosis. (**F**–**H**) GFAP immunoreactivity showing granular bodies and dense filamentous deposits. Astrocytic gliosis is particularly evident in the pons (**F**), medulla oblongata (**G**) and spinal cord (**H**) associated with swollen neurons. Images from patients who did not have previous neurological deficits. Scale bars: (**A**,**G**) = 50 µm; (**B**,**F**,**H**) = 100 µm; (**C**,**D**,**E**) =14 µm.

Neurons feltwork (neuropil) displayed a chaotic meshwork, with smaller dot-like structures and pyknotic neurons (Figure 4A–D). A spectrum of astrocytic pathology, highlighted by GFAP immunoreactivity, showed fibrillary gliosis (Figure 4E) and dense filamentous deposits (Figure 4F–H). The astrocytic reaction was particularly evident in the pons, medulla oblongata (Figure 4E–G) and spinal cord (Figure 4H), associated with swollen neurons or as a haphazard response to neuronal loss.

**Figure 5:**
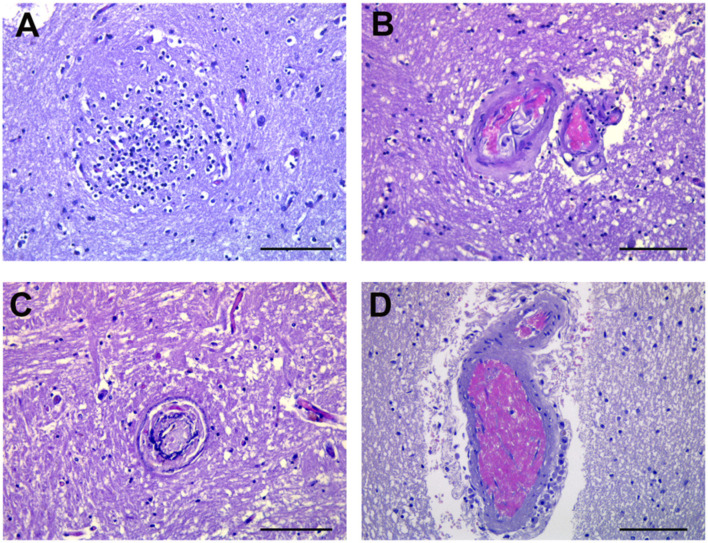
Pathologic reaction of glia and vascular changes. (**A**) Clusters of microglia (microglial nodules) mark the site of the destroyed neurons (arrows), and is the hallmark of neuronophagia. (**B**–**D**) Microangiopathy in most of the small vessels. Deposits of eosinophilic hyaline material with a “lumen within a lumen” appearance, siderocalcinosis and ferruginazation of microvessels and fibrosis are visible. Images from patients who did not have previous neurological deficits. Scale bars: (**A**–**D**) = 50 µm.

In 9 out of 10 cerebral cortex regions, neuronophagia was represented by an aggregation of microglia marking the site of destroyed neurons with a microglial nodule formation (Figure 5A). Microangiopathy, characterized by deposits of eosinophilic hyaline material of “lumen within a lumen” appearance, siderocalcinosis and ferruginazation of microvessels and fibrosis (Table 3) was diagnosed in cerebral cortex tissues from eight patients, and in the pons from five patients (Figure 5B–D).

### 3.4. Perivascular Inflammation

**Figure 6:**
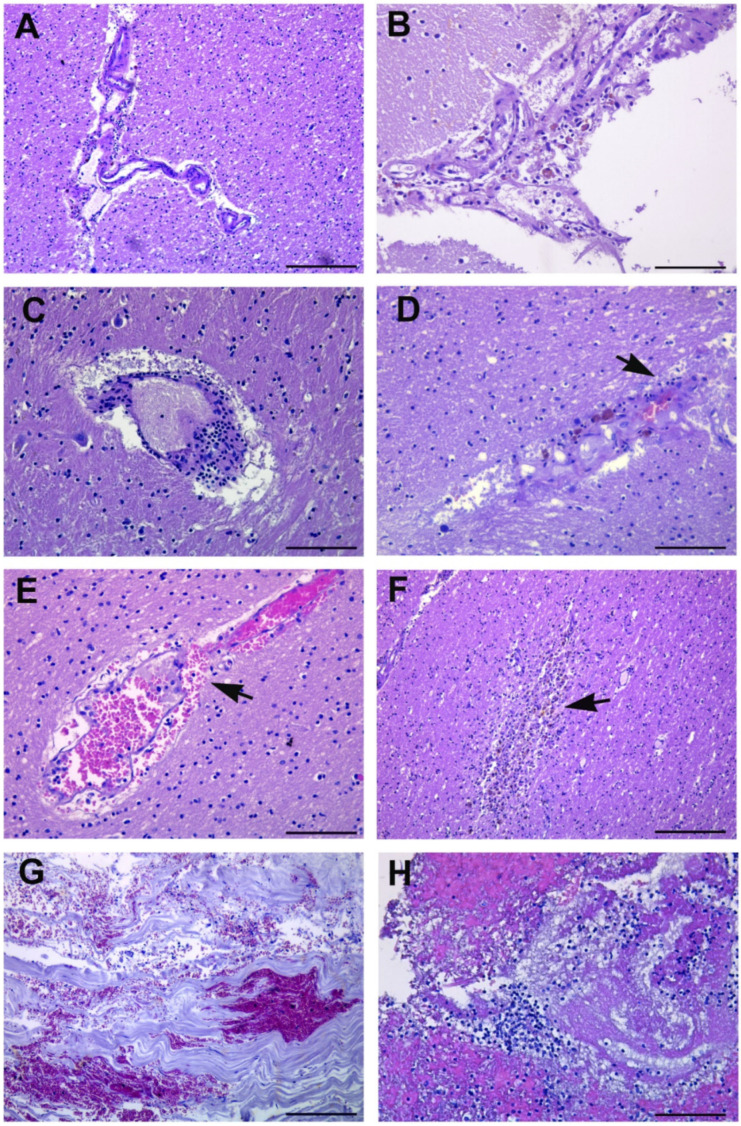
Brain histopathological findings. (**A**) Perivascular lymphocytic infiltrates the cortical parenchyma around the cerebral vessels. (**B**) Perivascular spread of inflammatory cells in the cerebellar cortex. (**C**) Basal nuclei. Perivascular cuffing by lymphocytes within the gray matter. (**D**) Cortical white matter section showing gliosis associated with a cluster of lymphocytes and macrophages around the blood vessel. Arrow points to siderophages. (**E**) Perivascular hemorrhage in the pontine region due to the rupture of the blood vessel wall (arrow) associated with the presence of inflammatory cells. (**F**) Necrotic cerebral cortex tissue (subacute-chronic cerebral infarction) (arrow) showing numerous infiltrating cells. (**G**,**H**) Meninges sections with hemorrhage and inflammation associated with necrosis. Images from patients who did not have previous neurological deficits. Scale bars: (**A**,**F**,**G**) = 100 µm; (**B**–**E**,**H**) = 50 µm.

Microscopic analysis showed lymphocytic inflammation targeting the vascular wall in the brain cortex (Figure 6A,D), cerebellum (Figure 6B), basal nuclei (Figure 6C) and brainstem, along with a perivascular hemorrhage due to a rupture of the blood vascular wall (Figure 6E).

In three patients, microscopic examination showed irregular zones of coagulative necrosis with marked cellular infiltration by foamy macrophages and reactive astrocytes around the necrotic focus, containing stick-like debris residual to either necrotic axons or vessels (Figure 6F).

In the cerebral cortex, hemorrhagic areas were found in the meninges of 8 out of 10 cases (Table 3; Figure 6G,H).

**Figure 7:**
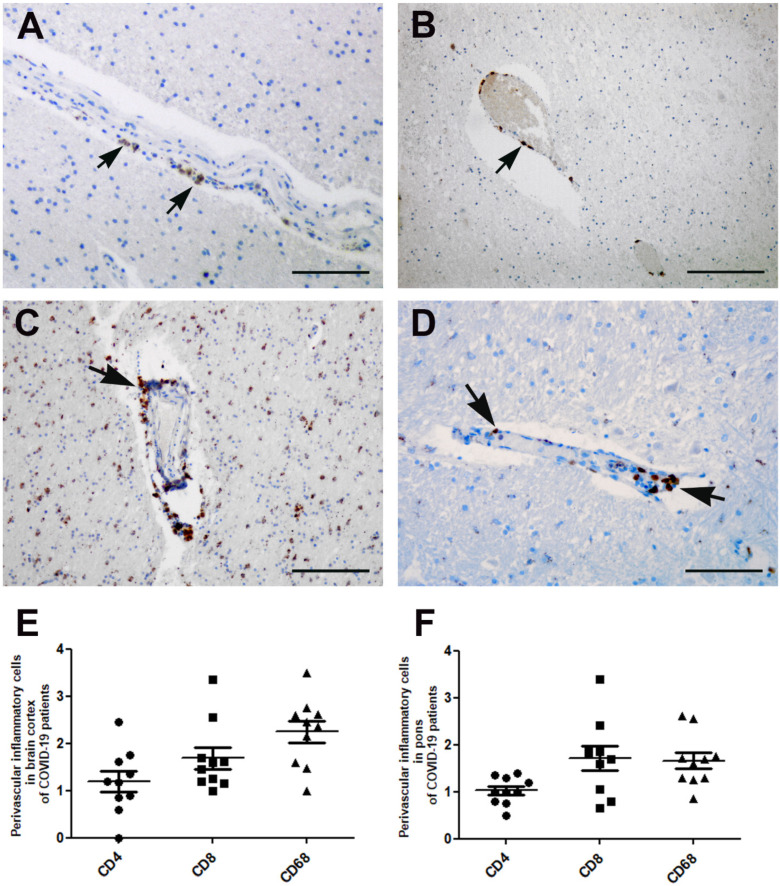
Inflammatory cells immune-characterization in the cerebral cortex and pons areas. (**A**) Lymphocytic inflammation in the cerebral cortex targeting the vessel wall showed the presence of CD4-positive lymphocytes (arrows) and (**B**) CD8-positive cells (arrow). (**C**) Numerous inflammatory cells are macrophages, as demonstrated with anti-CD68 immunohistochemistry in the cerebral cortex (arrow) and (**D**) pons area (arrows). (**E**) Mean number of perivascular inflammatory cells in the cerebral cortex and (**F**) pons area. Images from patients who did not have previous neurological deficits. Scale bars: (**A**,**D**) = 50 µm; (**B**) = 200; (**C**) = 100 µm.

Immunohistochemical characterization of perivascular inflammatory cells in the cerebral cortex and pons showed the presence of T lymphocytes, CD3 positive (Figure 7A), showing predominantly a CD8+ immunophenotype (Figure 7C), and less of a presence of CD4+ cells (Figure 7B). CD68 immunostaining highlights the presence of macrophages around small veins, arterioles and venules (Virchow Robin space) in cortical and subcortical regions within cerebral cortex parenchyma, as well as in the cerebellum (data not shown) and in cerebral cortex, mostly the pons (Figure 7D). In order to identify the cell types among the perivascular inflammatory cells, a quantitative analysis of CD4, CD8 and CD68 positive cells has been reported both in cerebral cortex (Figure 7E) and in the pons areas (Figure 7F).

## 4. Discussion

SARS-CoV-2 infection is known to cause multisystem disease and significant pathological changes in most organs [1]. Among a wide range of clinical presentations, neurological manifestations have been frequently observed in COVID-19 patients. Although the number of reports dealing with neurological complications are increasing, the suggested pathological mechanisms are varied and still inconclusive. It has been speculated that the neurological implications of COVID-19 could be the result of a direct virus neuroinvasion (though rarely proven), immune response or secondary to treatments and involvement of other organs.

Limited studies have described a post-mortem examination of the brain [5,6,7,12,13], and a wide range of histologic findings have been reported, also reflecting the heterogeneity of neurological complication in different patients. In addition, a non-standardized sampling procedure adds further heterogeneous results.

Here we described post-mortem macroscopic and microscopic alterations in 10 COVID-19 patients, who differed in sex, age, hospital stay and duration of the disease.

None of our patients presented with encephalitis or meningitis as the cause of death, and only one was one was diagnosed with stroke.

Some alterations of the cerebral cortex and cerebellum, as well as petechial hemorrhages within the white and gray matter, neuronophagia and microglial nodules occurred in all patients. These lesions have been previously described in the cerebral cortex of COVID-19 patients [14], however they represent hypoxic/ischemic changes. Indeed, oxygen deprivation is a primary causal factor of damage in the cortex and cerebrum, which is common in critically ill patients.

The cerebral brainstem is instead more resistant to oxygen deprivation [15]. In these areas we observed morphological lesions of neurons and neuropil in all patients. We identified alterations of neurofilaments scaffolding functions, with derangement of radial architecture of axons and formation of axonal spheroids as features of axonal damage. These findings are similar to the lesions seen in neurodegenerative diseases, such as Amyotrophic Lateral Sclerosis; Alzheimer’s disease; Parkinson’s disease [16]. Since the white matter of the brainstem carries most of the signals between the cerebral cortex and the spinal cord, and because the cranial nerve nuclei are located in the brainstem, which have a crucial role in vision, balance, hearing, swallowing, taste, speech, motor and sensory supply to the face [17], the lesions seen in this area may be responsible for some COVID-19-related neurologic symptoms [18].

Some studies described different grades of inflammatory infiltrates in the cerebral cortex parenchyma of COVID-19 patients, from severe and disseminated (ADEM)-like [19], to focal and mild infiltrates [20] have been described.

In our case series, we described multifocal lymphocytic inflammation, targeting the disrupted vascular wall in the analyzed areas. Immunocharacterization showed that the perivascular inflammatory infiltrate was mostly composed of CD8 positive T cells and CD68 positive macrophages. The presence of T lymphocytes and macrophages, associated with perivascular inflammation, supports the idea that microvascular injury can play a role in COVID-19 neurological manifestations. In agreement with this, recently published data have described microvascular injury and neuroinflammation with the activation of innate and adaptive immune cells in COVID-19 patients [9,10,21]. Histopathological findings of our case series could contribute to an increase in the current knowledge on the neuropathology of COVID-19 patients, which can lead to an adequate management of these patients.

## Figures and Tables

**Table 1:** Demographic, neurological history and clinical features.

N°	Age/Sex	Comorbidities	Neurological History	Neurological Symptoms at Admission	Neurological Symptoms During Hospitalization	Oxygenation/Intensive Care	Post-Mortem Gross Findings	Hospital Stay (Days)
1	90/F	IC; CKD;AF; partially-sighted	Lewy bodies dementia	Disoriented	Increasing somnolence	Oxygen nasal goggles	Left pneumonia; endocarditis; myocardial infarction	33
2	85/M	COPD	Dementia	Somnolence	Loss of consciousness	Oxygen therapy with VMK 50%	Chronic ischemic cardiopathy; heart failure; left pneumonia	16
3	70/F	Type II DM; hypertensive cardiopathy.	Bipolar disorder; pontine lesion	Somnolence	Balance deficit/cognitive impairment/nonresponsive to verbal stimuli.	Oxygen therapy with VMK 60%	Bilateral interstitial pneumonia; pleural and pericardial effusion; hypovolemic shock	72
4	60/M	Hypertension	None	None	None	cPAP/O2 therapy with VMK alternate; tracheostomy	Bilateral interstitial pneumonia; cardiogenic shock	33
5	57/M	Hypertension; DM;COPD	None	None	None	VMK; orotracheal intubation; tracheostomy	Bilateral interstitial pneumonia; acute pneumonia; heart failure;	14 + 27
6	68/M	Hypertension; cardiopathy; metabolic syndrome; autoimmune hypotiroidism; psoriatic arthritis	None	None	None	NIV; orotracheal intubation; ECMO	Bilateral interstitial pneumonia; acute pneumonia.	41
7	66/M	None	None	Somnolence Disoriented	diffuse brain deficiency/seizures/Coma	NIV; orotracheal intubation	DIC; hemorrhage of right atrium, endocardium, coronary sinus area	11
8	63/M	Hypertension; cerebral ischemic vasculopathy	Transient ischemic attack	Anosmia	Coma	Oxygen therapy with VMK 40%; orotracheal intubation	Interstitial pneumonia, DAD	5
9	38/M	Obesity (BMI 43)	None	None	None	Ventimask orotracheal intubation; VMK	Myocarditis; interstitial pneumonia, broncopneumonia	36
10	44/M	Sudden Death	None	None	None	No therapy	Myocarditis, pulmonary edema, demyelination of the pons and the medulla oblungata	Sudden death

IC = Ischemic cardiopathy; CKD = chronic kidney disease; AF = atrial fibrillation; COPD = chronic obstructive pulmonary disease; DM = diabetes mellitus; DIC = disseminated intravascular coagulopathy; NIV = not invasive ventilation; ECMO = extracorporeal membrane oxygenation; VMK = mechanical ventilation; cPAP = Continuous Positive Airway Pressure.

**Table 2:** Brain gross findings.

	Patient Number									
	1	2	3	4	5	6	7	8	9	10
Cerebral meninges alterations	Hyperhemic Leptomeninges Sub-dural hematoma	Hyperhemic Leptomeninges	Hyperhemic Leptomeninges	Hyperhemic Leptomeninges		Hyperhemic Leptomeninges	Hyperhemic Leptomeninges Sub-dural hematoma	Hyperhemic Leptomeninges	Hyperhemic Leptomeninges	Hyperhemic Leptomeninges
Cerebellum			Right lobe hemorragia				Right lobe hemorragia			
Hemorrhagic petechias							Right frontal lobe and basal nuclei	Pontine region	Basal nuclei	Basal nuclei
Other Changes	Hydrocephalus ex vacuo Multiple choroid plexus cysts	Hydrocephalus ex vacuo	Cerebral hemorrhage of right thalamic and pontine region		Cerebral edema	Cerebral infarction with hemorrhagic lesion of right occipito-parietal lobes with mesencephalic hemorrhage				

**Table 3:** Histopathological features.

PATIENT		1	2	3	4	5	6	7	8	9	10
**CEREBRAL CORTEX**	Perivascular Inflammation	++	++	++	++	++	++	++	++	++	++
	Meningeal Hemorrhage	++	++	++	+	+	++	++	++	-	-
	Parenchymal Hemorrhage	++	+	++	++	++	++	++	-	-	++
	Vascular Hyaline Wall and Mineral deposits	++	++	++ (focal)	++	++ (focal)	++	++	-	++	-
	Neuron Damage	++	++	++	++	++	-	++	++	++	-
	Neuronophagia	++	++	++	++	++	++	++	++	++	-
	Infarction	-	++	-	-	-	++	++	++ (focal ischemic change)	++ (hemorragic)	-
	Other Changes	Lewy bodies Arach-id cysts Ferrugi-us neurons	Pituitary fibrosclerosis	Basal ganglia and thalamus hemorragic necrosis				Acute inflammation neutrophil dominance	Dense meshwork of glial fibers	Pseudolaminar necrosis	
**CEREBELLUM**	Perivascular Inflammation	Colliquative degeneration	++	++	++	++	Colliquative degeneration	++	+	++	++
	Parenchymal Hemorrhage	Colliquative degeneration	-	++	-	-	Colliquative degeneration	++	-	++	-
	Loss Purkinjie Cells	Colliquative degeneration	++	++	++	++	Colliquative degeneration	++	-	++ (Pyknosis, red neurons)	++ (focal)
	Loss Granular Layer Neurons	Colliquative degeneration	-	++	++	++	Colliquative degeneration	++	-	++	-
**PONS**	Perivascular Inflammation	++	-	++	++	++	++	++	+	+	+
	Meningeal Hemorrhage	-	-	-	-	-	++		-	-	-
	Parenchymal Hemorrhage	++	-	++	-	++	++	++	++ (focal)	+ (perivascular)	++ (focal)
	Vascular Changes Hyaline Wall	++	-	++	-	-	++	++	+	-	-
	Neuron Damage	++	++	++	++	++	++	++	++	++	++
	Neuron Damage (NF aggregation *)	++	-	++	++	++	++	++	++	++	++
	Neuronophagia	++	++	++	++	++	++	++	++ (glial nodules)	++	-
	Derangement of neuropil and axonal swelling	++	++	++	++	++	++	++	++	++	++

NF * Neuronal intermediate filaments aggregation in perikarya. Histopathological score: - absent; + mild; ++ moderate.

## Data Availability

All relevant data are within the manuscript.

## Supplementary Materials

The following are available online at www.mdpi.com/article/10.3390/cells10092262/s1, Table S1: Laboratory findings, referred to the day before death, Table S2: ICU and non-ICU hospitalization and anticoagulant medications.
