# Supplementary material for: Neuropathology and Inflammatory Cell Characterization in 10 Autoptic COVID-19 Brains
Source: Cells. 2021 Aug 31;10(9):2262. doi: 10.3390/cells10092262 (PMC8469459; doi:10.3390/cells10092262)
Supplement: Supplementary file 1 [file cells-10-02262-s001.zip › cells-1342092-supplementary.pdf]

Table S1

Laboratory findings, referred to the day before death.

PATIENT N.	Gender	Age	Laboratory findings *				
			**Fibrinogen**	**D-Dimer**	**Lymphocyte**	**C-Recative protein**	**CPK**
**1**	F	90	150	2162	4.2	4.86	257
**2**	M	85	403	963	1.39	2.57	-
**3**	F	70	531	1519	9.2	4.44	231
**4**	M	60	666	4074	1.26	29	109
**5**	M	57	519	3892	1.40	1.14	215
**6**	M	68	-	-	-	-	-
**7**	M	66	537	968	0.48	3.12	72
**8**	M	63	726	1428	0.61	11.03	1313
**9**	M	38	749	1578	4.78	16.35	224
**10**	M	44	-	-	-	-	-

*Normal Values: Lymphoyte count x 10^{3} (1-3.5); D-Dimer ng/mL (0-500); Fibrinogen mg/dL (150-400); LDH U/L (200-400); PCR mg/dL (0.0 – 0.5); CPK U/L (52-336 M; 38-176 F)

Table S2

ICU and non-ICU hospitalization and anticoagulant medications.

PATIENT N.	Gender	Age	Clinical characteristics				
			ICU (days)	GCS score	Intubation/Sedation	Anticoagulant Home Therapy	Anticoagulant Hospital Therapy
**1**	F	90	-	-	-	-	Enoxaparin
**2**	M	85	-	-	-	Acetylsalicylic acid	Enoxaparin
**3**	F	70	1	12	-	-	Enoxaparin
**4**	M	60	27	15	Tracheostomy/ Sedation	-	-
**5**	M	57	26	N/A	Orotracheal intubation/ Sedation	Acetylsalicylic acid	N/A
**6**	M	68	40	N/A	Orotracheal intubation	-	N/A
**7**	M	66	10	15	Orotracheal intubation/ Sedation	-	Enoxaparin
**8**	M	63	2	3	Orotracheal intubation/ Sedation	-	Enoxaparin
**9**	M	38	24	15	Tracheostomy Orotracheal intubation/ Sedation	-	Enoxaparin
**10**	M	44	-	-	-	-	-

*****During Intensive Care Unit (ICU) stay.

GCS= Glasgow Coma Scale; Acetylsalicylic acid= Aspirin; Enoxaparin= Clexane 4000/6000; N/A= not available
